# The conundrum of the definition of haemorrhagic shock: a pragmatic exploration based on a scoping review, experts’ survey and a cohort analysis

**DOI:** 10.1007/s00068-022-01998-9

**Published:** 2022-06-22

**Authors:** Arthur James, Paer-Selim Abback, Pierre Pasquier, Sylvain Ausset, Jacques Duranteau, Clément Hoffmann, Tobias Gauss, Sophie Rym Hamada, for The Traumabase Group

**Affiliations:** 1 Department of Anaesthesiology and Critical Care, Pitié-Salpêtrière Hospital, Sorbonne University, GRC 29, AP-HP, DMU DREAM, 75013 Paris, France; 2 Department of Anesthesia and Critical Care, Hôpital Beaujon, Hôpitaux Universitaires Paris Nord Val de Seine, APHP, Clichy, France; 3 Department of Anaesthesiology and Critical Care, Percy Army Training Hospital, Clamart, France; 4 Val-de-Grâce French Military Medical Academy, Paris, France; 5 French Military Medical School, 69500 Bron, France; 6 Department of Anesthesiology and Intensive Care, Paris-Saclay University, Bicêtre Hospital, Assistance Publique Hôpitaux de Paris (AP-HP), Le Kremlin-Bicêtre, France; 7 Burn Center, Percy Military Teaching Hospital, Clamart, France; 8 CTB HIA Percy, 101 Avenue Henri Barbusse, BP 406, 92141 Clamart Cedex, France; 9 Department of Anesthesiology and Critical Care, Hôpital Européen Georges Pompidou, APHP, Université de Paris, 20 rue Leblanc 15, Paris, France; 10 CESP, Université Paris-Saclay, INSERM U10-18, Paris, France; 11 CESP, INSERM, Maison de Solenn, 97 boulevard de Port-Royal, 75014 Paris, France

**Keywords:** Severe trauma, Hemorrhagic shock, Outcome, Methodology, Scoping review, Experts’ survey

## Abstract

**Purpose:**

Traumatic hemorrhagic shock (THS) is a complex, dynamic process and, no consensual definition of THS is available. This study aims (1) to explore existing definitions of traumatic hemorrhagic shock (THS), (2) to identify essential components of these definitions and (3) to illustrate in a pragmatic way the consequences of applying five of these definitions to a trauma registry.

**Methods:**

We conducted (1) a scoping review to identify the definitions used for traumatic hemorrhagic shock (THS); (2) an international experts survey to rank by relevance a selection of components extracted from these definitions and (3) a registry-based analysis where several candidate definitions were tested in a large trauma registry to evaluate how the use of different definitions affected baseline characteristics, resources use and patient outcome.

**Results:**

Sixty**-**eight studies were included revealing 52 distinct definitions. The most frequently used was “a systolic blood pressure (SBP) less than or equal to 70 mmHg or between 71 and 90 mmHg if the heart rate is greater than or equal to 108 beats per min”. The expert panel identified base excess, blood lactate concentration, SBP and shock index as the most relevant physiological components to define THS. Five definitions of THS were tested and highlighted significant differences across groups on important outcomes such as the proportion of massive transfusion, the need for surgery, in-hospital length of stay or in-hospital mortality.

**Conclusions:**

This study demonstrates a large heterogeneity in the definitions of THS suggesting a need for standardization. Five candidate definitions were identified in a three-step process to illustrate how each shapes study cohort composition and impacts outcome. The results inform research stakeholders in the choice of a consensual definition.

**Supplementary Information:**

The online version contains supplementary material available at 10.1007/s00068-022-01998-9.

## Introduction

Traumatic Hemorrhagic Shock (THS) remains the leading cause of preventable death [1], however no universal definition is available. A recent work attempted to establish a Delphi-based consensus for massive transfusion [2]. The trauma literature of the last decade applied a multitude of definitions to this condition. Most often, these definitions used varying combinations of blood pressure, heart rate, shock index or transfusion-related criteria. Several definitions integrated a time-dependant component such as blood product use over time which is supposed to reflect the dynamic nature of THS [3–5]. This heterogeneity results from a complex pathophysiology and differing conceptions and treatment doctrines. In consequence, cohorts are difficult to compare, therapeutic targets and outcome definitions vary widely, leading to inconclusive results and a potential waste of a precious research resource [6].

A standardization of the THS definitions could accelerate the identification and treatment of patients in shock. It could also provide clarification for observational and interventional research. In analogy, the establishment of a consensus definition of sepsis and septic shock was a major step forward in the fight against this condition [7, 8]. Considering THS management as a complex intervention and dynamic process [9, 10], it appeared appropriate to explore existing definitions of THS and evaluate their clinical relevance.

The objective of the present study was not to provide a definitive definition, but to perform an exploration and comparison of existing definitions and identify and trial a number of candidate definitions in order to prepare and inform a future consensual, international effort. For this reason, the present study included (1) a scoping review of the literature to identify existing definitions of THS, (2) a survey of international experts to explore the importance given to different possible components of the THS definition and (3) a cohort study to evaluate how the use of different definitions of THS might affect diagnosis, patient management and outcomes.

## Materials and methods

This study consisted of three steps: (1) a scoping literature review, (2) an international online survey and (3) a register-based study.

### Scoping literature review

A scoping literature review was conducted to identify THS definitions used in the trauma literature and to circumscribe the components of THS [11]. Scoping reviews are form of knowledge synthesis that addresses an exploratory research question aimed at mapping identify knowledge gaps, scope a body of literature or clarify concepts [12, 13]. A search was conducted from January 2010 to November 2018 (included) in *MEDLINE* (via PubMed) and *Google Scholar* using a controlled vocabulary (Supplementary material 1) without language restriction. In both databases, the study group investigated the 100 first responses (respectively using *best match* and *by relevance* ranking). The *clinicaltrials.com* database was explored using the same keywords. To broaden this initial search, (1) all the authors (AJ, PSA, PP, SA, CH, TG, SH) were asked to share articles that might include a THS definition and (2) all respective bibliographies were hand-searched using a snowballing method [14]. All published articles that included major trauma patients and provided a definition related to traumatic THS were considered for inclusion. All types of published articles were considered and included: randomized control trials (RCT), observational studies (prospective or retrospective), editorials and protocols. Technological assessments, non-human studies, cost-effectiveness assessments were excluded. In cases of multiple publications of the same study, the most up-to-date or comprehensive publication was included.

Authors (AJ, CH, PP, PSA) identified and extracted from original articles the literal definitions applied to define a THS. They also extracted whether the THS definition was supported by a reference or not.

### Online experts survey

The survey was developed in a three-step process. First, two members of the working group (AJ, SH) conceived a questionnaire including all components identified from the scoping literature review. Second, all working group members (AJ, PSA, PP, SA, CH, TG, SH) reviewed the questionnaire. Third, a convenience sample of ten international trauma experts for clarity and capacity to provide meaningful answers. After each step, the working group improved the survey according to the provided feedback.

Then, a convenience sample of 64 international experts were selected based on their contribution to major publications about THS (such as guidelines and highly cited publications authors) or identified as opinion leaders (such as conferences speakers) and invited personally to reply to the online survey (www.surveymonkey.com). At least three individual e-mail reminders were dispatched to ask for a response. As the survey was based on voluntary participation and information disclosure, the study protocol did not undergo review by an institutional review board. Voluntary participation was considered as consent. The acknowledgments lists all experts who accepted to be named.

The survey consisted of 50 questions organised in three sections: (1) expert profile (country of practice, main clinical area of practice, context (civilian or military), number of personal publications related to the subject), (2) selection of *components* of THS among clinical & biological components, scores and transfusion-related parameters and (3) conclusion. The ranking of selected *components* from step 2 ranged from − 3 (*I strongly disagree with the importance of this component*) to + 3 (*I strongly agree with the importance of this component*) with 0 being neutral. Components with a median scoring [1;3] were considered as *consensual*, those scoring between [− 1;1] as *neutral* and those scoring [− 3;− 1] as *non-relevant *[15]*.* For numerical components, experts were requested to provide a threshold on an analogue scale.

### Registry-based study

Based on the scoping review and on the results from the expert survey, the working group identified consensus and operational definitions of THS. Then, these definitions were applied to a large register of trauma patients, to evaluate how the different definitions affected diagnosis, management and patient outcome. This population was extracted from the Traumabase Registry (www.traumabase.eu) from January 2010 (registry initiation) to November 2018 (date of data extraction). The Traumabase registry includes all consecutive patients admitted in all participating level-1 trauma centers for suspected major trauma based on the national Vittel triage criteria [16, 17]. These centers provide the highest level of care for trauma patients in their respective geographical areas. The Traumabase registry samples epidemiological, prehospital, resuscitation, critical care and outcome data. Data collection is performed by trained clinicians and research assistant under supervision of a designated coordinator in each center. The registry database on a secure server, contains numerous consistency and internal validation algorithms. Data management is performed by professional data managers and statisticians from the Research Unit Paris 7. All 14 centers including patients at the time of data extraction participated in the study.

Definitions were translated step by step by two investigators (AJ, SH) to correspond to a mathematical script to classify patients within the data set accordingly (Supplementary material 2).

For each of the five definitions, a THS population was identified and described in terms of:

- Baseline characteristics: general parameters (age, sex, mechanism of injury); on-scene parameters and actions (Glasgow Coma Scale, cardiac arrest, physiological parameter, intubation); resuscitation room parameter and diagnostic explorations (basics physiological parameters, Focused Assessment Sonography for Trauma (FAST) echography); biological parameters (Blood lactate, prothrombine time (PT), fibrinogen, haemoglobin, platelet); severity scores (Simplified Acute Physiology Score (SAPS2), Sepsis-related Organ Failure Assessment (SOFA) [18], Injury Severity Score (ISS) [19], Trauma Injury Severity Score (TRISS) [20] and injury location (based on the Abbreviated Injury Scale (AIS) classification) [21].
- Resource use: blood product requirement (blood products volumes in trauma bay and at 24 h), rate of massive transfusion (defined as ten or more RBC in the first 24 h), interventional procedure requirement (surgery, interventional radiology) needs, Intensive Care admission (ICU) Length Of Stay (LOS) and in-hospital LOS.
- Outcomes: episode of renal failure (Renal Replacement Therapy [RRT], Acute Respiratory Distress Syndrome [ARDS] based on Berlin criteria) and mortality (First 24 h and in-hospital).

### Statistical analysis

Quantitative data were described with median and interquartile range [Q1; Q3] and categorical data with counts and percentage (%). A sensitivity analysis examined apart the RCTs. All analysis involved the use of R v4.0.2 (www.R-project.org, the R foundation for statistical Computing, Vienna, Austria).

## Results

### Scoping literature review

Among the 200 articles screened on MEDLINE and *Google Scholar* and those highlighted by the snowballing, the scoping review identified 68 studies that involved a definition of THS (44 published articles identified in *MEDLINE* or *Google Scholar* and 24 protocols identified in www.clinicaltrials.gov) used whether as an inclusion criterion or as an outcome (Supplementary material 3). Among these 68 definitions, 52/68 (76%) were distinct and stand-alone (Supplementary material 3) resulting in only 5 distinct definitions used in more than one article. Among the 44 published articles, 20 (45%) authors provided a reference to the used definition.

The most recurrent definition of THS (*n* = 12/68) was the presence of “a systolic blood pressure (SBP) less than or equal to 70 mmHg or between 71 and 90 mmHg if the heart rate is greater than or equal to 108 beats per min”. Cross-referencing several studies applying this definition identified randomized controlled trial by Bulger et al. on Out-of-hospital Hypertonic Resuscitation After Traumatic Hypovolemic Shock as the initial source for this definition [22]. SBP was involved in 48/68 (71%) definitions, with a systolic pressure threshold ranging from 70 to 120 mmHg. The most frequently used threshold for SBP was 90 mmHg (*n* = 41/68, 60%). Red blood cell (RBC) transfusion was involved in 17/68 definitions (25%), with very heterogeneous volume and time thresholds. Serum lactate concentration was involved in 8/68 different definitions (12%) with a threshold ranging from 2 to 5 mmol/L. 5/68 definitions (7%) applied the Advanced Trauma Life Support (ATLS) classification of hypovolemic shock (with threshold ranging from grade 1 to 3 when a threshold was specified).

A sensitivity analysis focusing only on the nine included RCTs, demonstrated that six of them (67%) referred to a unique definition (the so-called *Most Cited definition*) and four (44%) of them provided a reference to the used definition.

### Expert online survey

The responses to the survey were obtained between November 2018 and February 2019. Among the 64 experts invited to take part in the survey, 29 (45% response rate) from 10 different countries responded (France, *n* = 8; United Kingdom, *n* = 4; United states of America, *n* = 4; Germany, *n* = 4; Austria, *n* = 2; Switzerland, *n* = 2; Norway, *n* = 2; Israel, *n* = 1; Algeria, *n* = 1; Belgium, *n* = 1). These experts shared miscellaneous background with 8/29 (28%) practising mainly in prehospital care, 13/29 (45%) in intensive care, 11/29 (38%) in operating the, 7 (24%) in the emergency department, 6/29 (20%) in surgery and 3/29 (10%) in a laboratory (with some experts reporting multiple categories). Of these 21/29 (72%) experts were affiliated to civilian care; 2/29 (7%) to military care only and 6/29 (21%) were affiliated both to civilian and military care. The survey participants had a high level in expertise in trauma care with 11/29 (38%) reporting more than 20 publications as first author on trauma, 8/29 (28%) that report between 10 and 20 publications, 7/29 (24%) that report 5 to 10 publications and 3/29 (10%) that report less than five publications.

**Fig. 1:**
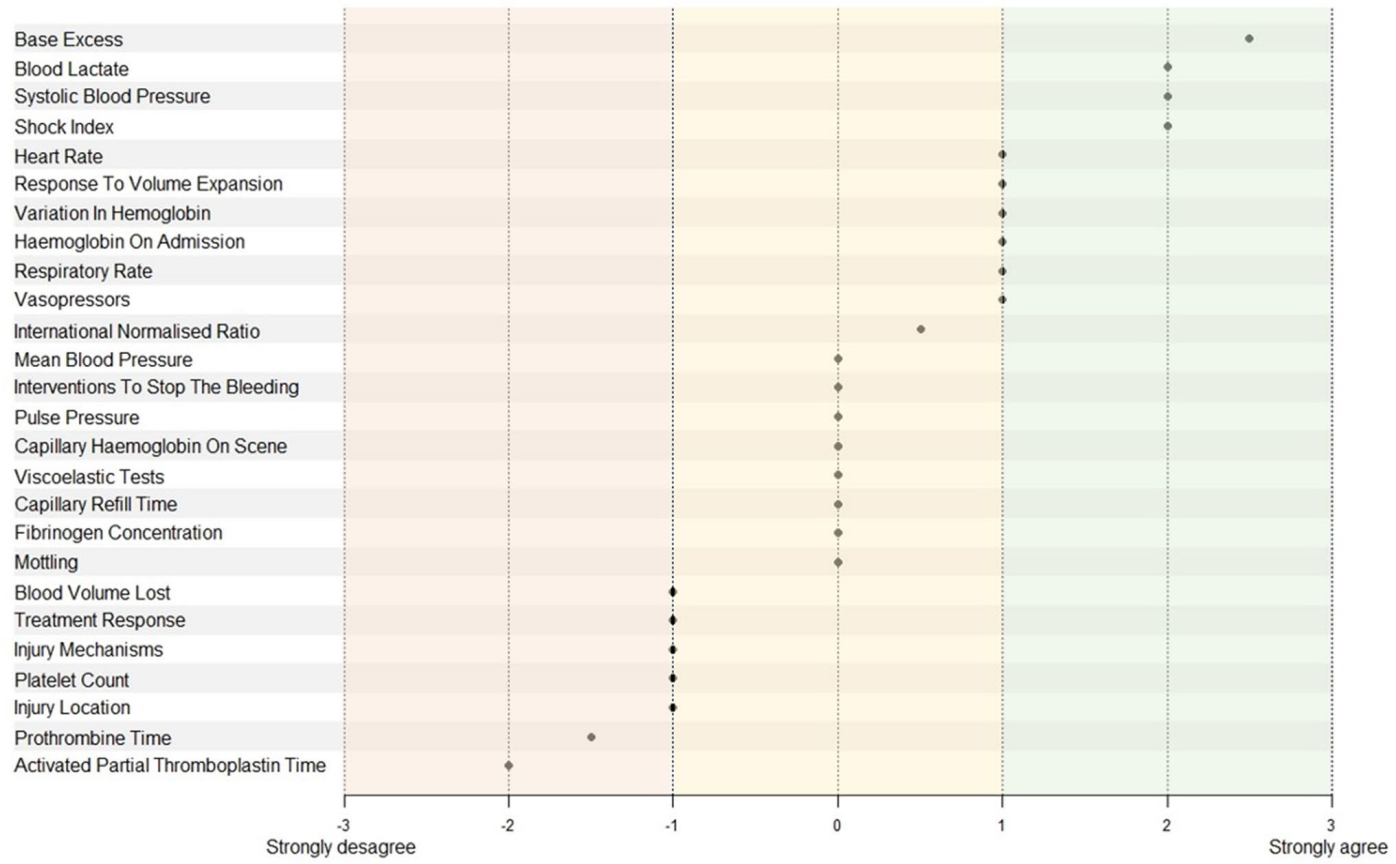
Expert scoring of variables, Parameters are presented as the median score of the 29 experts’ responses

The survey findings are summarized in Fig. 1 and Supplementary material 4 provides the median values and thresholds proposed by the experts.

The survey identified four consensual components of a THS definition (mean score [1–3]):

- base excess with a median expert score of 2.5 and a threshold of − 5 mEq/L
- blood lactate concentration with a median expert score of 2.0 and a threshold of 3 mmol/L,
- SBP with a median expert score of 2.0 and a threshold of 90 mmHg,
- shock index with a median expert score of 2.0 and a threshold of 1.

The survey identified 16 components with a lower level of consensus (mean score [0–1]): response to fluid challenge, variation in hemoglobin, hemoglobin at hospital, mean blood pressure, respiratory rate, intervention needed to stop the bleeding, pulse pressure, use of vasoactive drugs, hemoglobin on scene, viscoelastic tests, capillary refill time, fibrinogen, blood lost volume, mottling, treatment response, INR and five non-consensus components (Injury mechanism, platelet, injury location, Prothrombin Time and Activated Partial Thromboplastin Time) were also identified.

### Registry-based study

Based on the review and survey results, the study group purposively selected five definitions of THS (Box 1):

- the PROPPR study definition [23],
- the PROMMTT study definition [4],
- the Traumabase definition [5, 24–26],
- the “Most frequently cited” definition: “a systolic blood pressure (SBP) inferior or equal to 70 mmHg or between 71 and 90 mmHg if the heart rate is superior or equal to 108 beats per min”,
- the “Expert based definition” composed of the most consensual components provided by the 29 experts: *base excess* and *shock index*. We kept these two, because the shock index includes the SPB and because the *base excess* is strongly correlated with the *pH*. Based on experts’ responses, we used − 5 mEq/L and 1.0 as thresholds for base excess and shock index respectively (Supplementary material 4).

**Box 1:** THS definitions

Most frequently cited definition
SBP ≤ 70 mmHg
OR
SBP 71–90 mmHg if heart rate ≥ 108/bpm
Expert definition
Shock Index ≥ 1.0 during prehospital care or in resuscitation room
AND
Based excess at hospital arrival ≤ -5.0 mmol/L
PROPPR study
> 1 RBC during prehospital stage or in resuscitation room
AND
ABC score [5] ≥ 2:
Penetrating mechanism (gunshot, stabbing)
Positive focused assessment sonography for trauma (hemoperitoneum)
SBP at hospital arrival ≤ 90 mmHg
Heart rate at hospital arrival ≥ 120 bpm
PROMMTT study
At least 1 RBC within 6 h
The Traumabase definition
At least 4 RBC within the first 6 h

Supplementary material 2 present the detailed components used in the database to categorize patients in groups

*RBC* red blood cell, *SBP* systolic blood pressure

These five definitions were applied in a cohort of 21,807 trauma patients from the Traumabase registry. Five cohorts of THS were identified according to the chosen definition: PROPPR (*n* = 881, 4.0%), PROMMTT (*n* = 2510, 11.5%), Traumabase (*n* = 1765, 8.1%), Expert-based definition (*n* = 2738, 12.6%) and the “Most Frequently Cited” definition (*n* = 3527, 16.2%).

These five THS populations were compared for baseline characteristics (Table 1) and outcomes (Table 2) resulting in important differences across groups, including the proportion of massive transfusion (ranging from 19.5% with the “most frequently cited” definition to 43.0% with the Traumabase definition), the proportion of patients requiring surgery within the first 24 h (ranging from 54.6% with the “most cited definition” to 75.0% with the Traumabase definition), in-hospital mortality (ranging from 27.7% with the experts definition to 37.1% with PROPPR definition), in-hospital length of stay (ranging from 10 days with the most cited definition to 17 days with the Traumabase definition).

**Table 1:** Baseline characteristics

	Complete sample (*n* = 21,807)	PROPPR (*n* = 881)	PROMMTT (*n* = 2510)	Traumabase (*n* = 1765)	Expert (*n* = 2738)	Most cited (*n* = 3527)
General characteristics						
Age (year)	36 [25; 53]	34 [24; 51]	39 [26; 56]	38 [25; 55]	41 [25; 54]	40 [26; 56]
Sex (men)	16,667 (77.9)	628 (71.6)	1743 (69.8)	1258 (71.6)	1857 (71.8)	2580 (73.5)
Mechanism (penetrating)	2502 (11.7)	214 (24.3)	380 (15.2)	274 (15.5)	336 (12.3)	432 (12.3)
On-scene parameters						
Glasgow coma score	15 [13; 15]	13 [4; 15]	14 [5; 15]	14 [5; 15]	14 [6; 15]	11 [3; 15]
Cardiac arrest	769 (3.7)	147 (17.1)	348 (14.1)	257 (14.8)	278 (12.3)	769 (22.1)
SBP (mmHg)	128 [111; 142]	98 [75; 120]	103 [80; 125]	100 [80; 121]	108 [88; 123]	104 [79; 128]
Heart rate (b/mn)	88 [75; 103]	119 [90; 131]	101 [80; 122]	105 [80; 125]	110 [90; 128]	100 [75; 120]
Shock index	0.81 [0.66; 1.00]	1.58 [1.27; 1.97]	1.32 [1.00; 1.69]	1.37 [1.04; 1.74]	1.00 [0.80; 1.26]	1.43 [1.15; 1.74]
Capillary haemoglobin concentration (g/dL)	14 [12.7; 15.0]	12.2 [10.5; 13.9]	12.3 [10.9; 14.0]	12.3 [11.0; 14.0]	13.0 [11.3; 14.3]	13 [11.0; 14.10]
I ntubation	5533 (26.2)	595 (68.4)	1486 (59.9)	1059 (60.7)	1600 (58.8)	2314 (66.0)
Admission to resuscitation room						
SBP (mmHg)	126 [110; 142]	86 [65; 110]	99 [75; 121]	96 [71; 120]	99 [80; 120]	99 [75; 124]
Heart rate (/mn)	87 [74; 101]	120 [95; 135]	103 [81; 121]	105 [85; 123]	108 [90; 123]	100 [79; 120]
Shock Index	0.7 [0.6; 0.8]	1.3 [1.0; 1.7]	1.0 [0.8; 1.4]	1.1 [0.8; 1.5]	1.1 [0.8; 1.4]	1.0 [0.7; 1.4]
FAST echography Abnormal (if done)	3420 (16.5)	599 (68.9)	1096 (45.2)	819 (47.9)	1090 (40.8)	1195 (35.1)
Biological parameters on admission to resuscitation room						
Haemoglobin (g/dL)	13.2[11.7; 14.4]	9.4 [7.8; 11.1]	9.8 [8.2; 11.4]	9.5 [7.9; 11.1]	11.0 [9.0; 12.8]	11.0 [9.1; 12.80]
Prothombin time (%)	84 [71; 94]	49 [33; 63]	55 [39; 69]	51 [36; 65]	62 [45; 77]	63 [45; 79]
Platelet (10^3^/mm^3^)	225 [184;26]	175 [122;23]	181 [130;23]	174 [122;22]	206 [153; 254]	201 [151;25]
Lactates (mmol/L)	2 [1.3; 3]	5.4 [3.2; 9.1]	4.1 [2.3; 7.4]	4.7 [2.6; 8.3]	4.0 [2.5; 6.4]	3.6 [2.1; 6.7]
Base excess (mEq/L)	− 3.2 [− 5.8; − 1.1]	− 10.8 [− 15.3; − 7.0]	− 8.6 [− 13.3; − 5.4]	− 9.5 [− 14.3; − 5.9]	− 8.6 [− 12.0; − 6.5]	− 7.5 [− 11.9; − 4.4]
Severity scores						
SAPS2	20 [12; 38]	55 [38; 73]	50 [36; 68]	52 [38; 70]	32 [47; 63]	50 [34; 68]
SOFA score	1 [0; 6]	10 [7; 13]	9 [6; 12]	10 [7; 13]	9 [5; 12]	9 [5; 12]
ISS	13 [6; 24]	32 [20; 43]	29 [18; 41]	29 [20; 43]	27 [17; 38]	25 [16; 38]
Predicted mortality	11.9 (23.7)	42.3 (37.5)	34.5 (47.6)	35.8 (48.0)	32.0 (34.3)	37.5 (37)
Injury patterns						
Severe head trauma	5864 (27.6)	298 (34.6)	888 (36.0)	610 (34.8)	1078 (39.8)	1434 (41.5)
Severe injuries location						
Head & neck	7019 (32.2)	378 (42.9)	1080 (43.0)	723 (41.0)	1233 (45.0)	1729 (41.5)
Chest	7016 (32.2)	541 (61.4)	1391 (55.4)	1010 (57.2)	1549 (45.0)	1851 (52.5)
Abdominal	3314 (15.2)	438 (47.9)	922 (36.7)	735 (41.6)	881 (32.2)	980 (27.8)
Extremities	5467 (25.1)	455 (51.6)	1319 (52.5)	1004 (56.9)	1231 (45.0)	1365 (38.7)

Variables are reported as median [IQR1, IQR3] or *n* (%); Severe injuries location is based on an AIS score ≥ 3; Severe head trauma is defined as documented injury on computer tomography scanner. Predicted mortality is based on the mean (Sd) TRISS

*SBP* Systolic Blood Pressure, *FAST* Focused assessment with sonography in trauma, *SAPS2* Simplified Acute Physiology Score 2, *SOFA score* Sequential Organ Failure Assessment score, *ISS* Injury Severity Score

**Table 2:** Clinical resource use and outcome components

	Complete sample (*n* = 21,810)	PROPPR (*n* = 881)	PROMMTT (*n* = 2,510)	Traumabase (*n* = 1,765)	Experts (*n* = 2,738)	Most cited (*n* = 3,527)
Transfusion in resuscitation room						
RBC	0 [0; 0]	3 [2; 4]	2 [1; 4]	2 [0; 4]	0 [0; 2]	0 [0; 2]
FFP	0 [0; 0]	0 [0; 3]	0 [0; 2]	0 [0; 3]	0 [0; 0]	0 [0; 0]
Platelets	0 [0; 0]	0 [0; 0]	0 [0; 0]	0 [0; 0]	0 [0; 0]	0 [0; 0]
Transfusion at 24 h						
RBC	0 [0; 1]	8 [4; 14]	6 [4; 12]	8 [6; 14]	4 [0; 9]	3 [0; 8]
FFP	0 [0; 0]	6 [2; 10]	4 [2; 8]	6 [3; 10]	2 [0; 6]	0 [0; 5]
Platelets	0 [0; 0]	1 [0; 2]	0 [0; 1]	1 [0; 2]	0 [0; 1]	0 [0; 1]
Massive transfusion	895 (4.6)	323 (40.6)	744 (32.5)	700 (43.0)	594 (23.9)	622 (19.5)
Tranexamic acid	4602 (26.3)	706 (83.4)	1,633 (73.5)	1,176 (76.2)	1,525 (63.0)	1,770 (57.2)
Fibrinogen concentrate	1005 (5.7)	392 (46.5)	816 (36.8)	644 (41.7)	635 (26.3)	697 (22.6)
Surgery/IR						
Immediate	849 (4.8)	243 (28.5)	413 (18.4)	351 (22.3)	292 (11.9)	371 (11.9)
within 24 h	10,128 (46.4)	589 (66.9)	1,754 (69.9)	1,323 (75.0)	1,733 (63.2)	1,924 (54.6)
Mortality						
24 h	721 (3.6)	150 (18.2)	356 (15.0)	254 (15.1)	286 (11.1)	499 (15.1)
In-hospital	2,182 (11.0)	305 (37.1)	818 (34.5)	604 (35.8)	711 (27.7)	1,190 (36.0)
Organ failure						
ARDS	1,252 (12.6)	142 (26.1)	383 (23.6)	314 (26.0)	443 (24.8)	518 (24.0)
RRT	302 (2.8)	71 (11.5)	170 (9.4)	153 (11.5)	175 (8.9)	182 (7.4)
LOS						
In hospital	8 [3; 19]	14 [2; 38]	16 [3; 40]	17 [2; 42]	16 [4; 38]	10 [2; 32]
In ICU	3 [2; 7]	6 [2; 18.75]	6 [2; 17]	6 [2; 18]	6 [2; 18]	5 [2; 15]

Variables are reported as median [IQR1, IQR3] or *n* (%); Massive transfusion is defined as more than 10 RBC in the first 24 h;

For each of the five definitions of THS, the number of patients selected using a given definition is presented in the first line of the table. As example, using the PROPPR definition would have conducted to identify among the 21,810 of the complete sample 881 as being in THS

*RBC* red blood cell, *FFP* fresh frozen plasma, *IR* interventional radiology, *ARDS* acute respiratory distress syndrome, *RRT* renal replacement therapy, *LOS* length of stay, *ICU* intensive care unit

## Discussion

### Main findings

The objective of this study was not to provide a definitive definition of THS, but provide substance to advocate for a concerted, international effort to define haemorrhagic shock and to inform and prepare this process. The three-step exercise identified five candidate definitions and demonstrated when applied to a large trauma data set how they generate considerably divergent study cohorts in terms of need for resource use and patient outcome including mortality.

Comparing the effect of the five candidate definitions, the groups appeared similar in terms of basic characteristics such as age, sex, mechanism, proportion of head injury as important confounder for overall mortality and AIS composition. All five candidate definitions seemed able to target groups of severe patients with SAPS 2 ranging from 32 (*expert*) to 55 (*PROPPR*), ISS ranging from 25 (*Most Cited*) to 32 (*PROPPR*), organ failure and high predicted and observed mortality for all groups. All definitions selected patients with severe physiological derangement with a Shock Index of 1 and above (1.3 for *PROPPR*), haemoglobin < 12 g/dl, Lactate > 3 mmol/l and BE > 7. The two definitions by far generating the most intense resource use and blood product consumption use on admission and at 24 h are consistently *PROPPR* and *Traumabase*. There indicators correlate well in these groups with marker of physiological derangement and patient outcome. The five candidate definitions were more or less selective, *PROPPR* being the most selective and the *Most Cited* recruiting the largest group but with low severity.

The authors struggled to find a reference for the *Most cited* definition; none of the published articles that applied it provided an original reference and the definitions seems not derived on any clinical cohort. It can probably be considered as outdated and be replaced by studies based on more rigorous and prospective approaches [27].

**Fig. 2:**
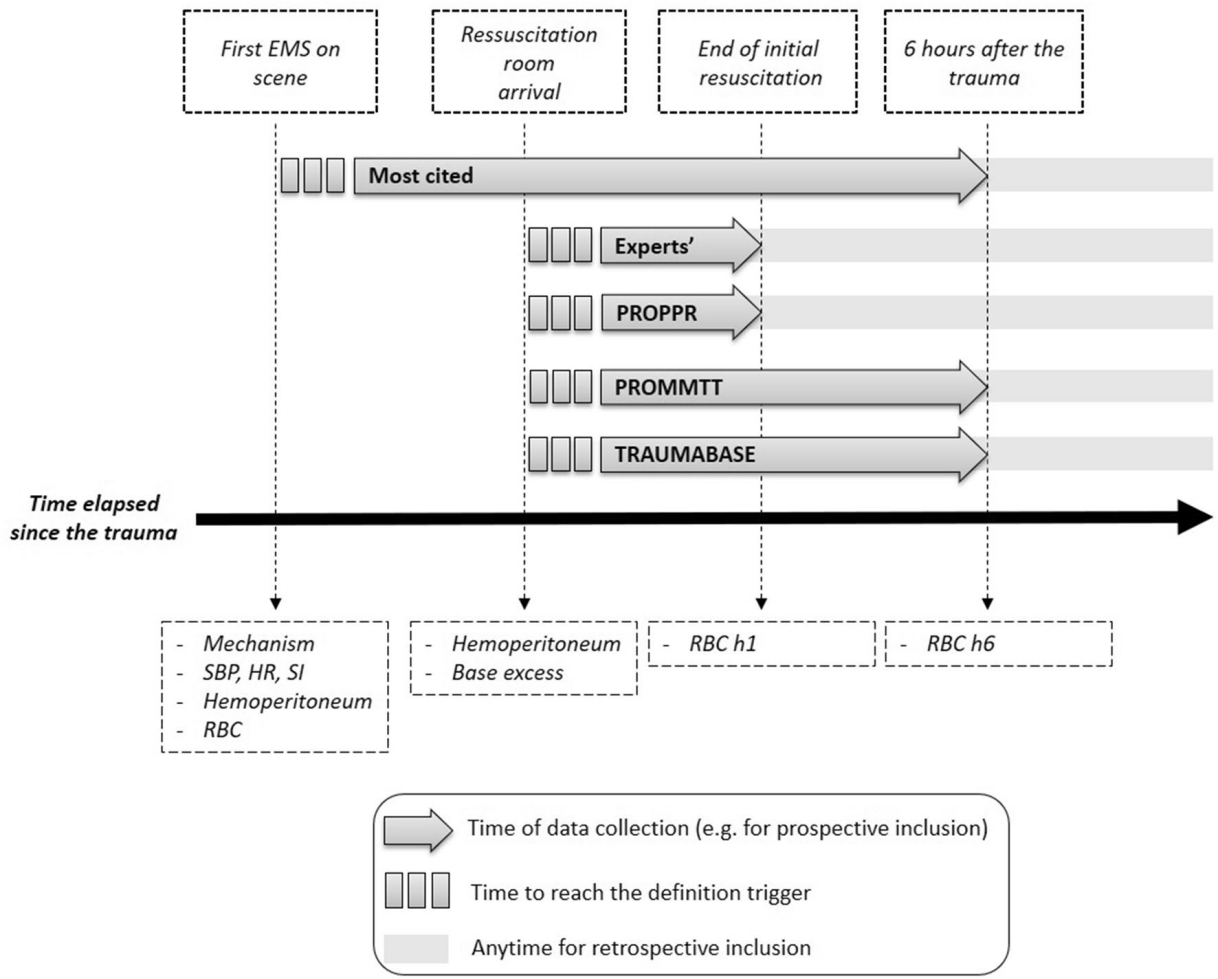
Timeline of definitions' availability. *EMS* emergency medical services, *SBP* systolic blood pressure, *HR* heart rate, *SI* shock index, *RBC* red blood cell, *PROPPR study* Pragmatic, Randomized Optimal Platelet and Plasma Ratios Study, *PROMMTT study* The prospective, Observational, Multicenter, Major Trauma Transfusion

Figure 2 illustrates the time dependency of each definition related to the patient pathway and how this relationship to time may influence the identification of patients in shock to prompt expedient treatment and/or the inclusion into a study protocol or observational cohort. The *Most Cited* can be applied on scene, all other definitions at the earliest in the resuscitation room or require a few hours to elapse. All take into account the fact that the early hours are essential.

To our knowledge, only one recent work addressed the challenge to derive a consensual definition for massive transfusion, a proxy for hemorrhagic shock, through an international expert Delphi process [2]. The experts voted for the following definition: 4 or more units of multiple blood components within 2 h of injury. This definition was unfortunately not available when the present work was under way. The definition is applicable within two hours of injury and resource based using all types of blood products but entails no physiological parameters. Excluding physiological data may omit patients initially in shock but stabilised through expedient medical and/or surgical intervention or die from shock on admission. A definition requiring a certain amount of blood products may discount patients that stabilised with smaller amounts, a recently observed tendency [28]. We applaud the work of Wong et al. and share their approach as the way forward to find a consensual definition. However, as the authors state themselves, the composition of their expert group is over-representative of North America and the UK and more suitable for systems with similar transfusion practice. Neither the definition used by Wong et al., nor any of the five candidate definitions explored in the present study includes an element of haemorrhage control resource need other than blood products. An aspect that may require further exploration. The international trauma community needs to take into account definitions for resource poor settings.

### Implications

Clinical research relies on a concise delineation of inclusion or outcomes criteria [7]. The use of poorly delineated definitions is likely to affect results by reducing reproducibility either in randomised controlled trials or in epidemiologic studies, with a risk to waste precious research resource and divert patient recruitment [29]. The present results demonstrate the existing heterogeneity in the available literature and explain the challenge to perform meaningful systematic reviews [30–32] and meta-analysis [33]. In consequence, a consensual international definition of THS is necessary.

In the present study, authors were careful to avoid recommending a single definition. But the results inform the inevitable and necessary future international consensus with precious insight. In agreement with the definition provided by Wong et al., a future THS definition should focus on the first hours of shock management to acknowledge the time-dependant and dynamic nature of THS and integrate consumption of all blood type of blood products. Such definition would also need to reflect that the later the THS is defined, more information (physiological, clinical, therapeutic) is available to make the diagnosis more specific. THS can indeed present with various presentations depending on the timeline. For example, immediately after the trauma a patient can present a significant bleeding from extremity injury with normal hemodynamic parameters and still functional physiological compensation. Despite hemorrhage control after application of a tourniquet, the same patient might then become hypotensive because of strained physiological compensation. A blood transfusion might thus be required to restore hemodynamic. In addition, a composite definition should include physiological parameters to not omit patients that stabilise or die early and expressed by the expert consensus. The inclusion of haemorrhage control interventions should be considered [27]. As demonstrated by the registry analysis in this work, the application of a specific definition has measurable impact on the composition of either prospective or retrospective study cohorts, patient outcome, resource use, the detection and diagnosis and in consequence the treatment of THS. A very inclusive definition that assures large recruitment, may not be ideal to identify bleeding patients that will require haemorrhage control and vice versa. Ideally, all these aspects require to be taken into account to find an international consensus. The example of the five candidate definitions show that several future candidate definitions need to be tested in large international cohorts to reach a final consensus.

### Limitations

This work also presents several limitations. First, our review is not a systematic review. However, scoping reviews are now endorsed by the PRISMA collaboration group [11] and the purpose of the study matches with a scoping review extent (clarify definition, explore breadth and map evidence) [34–36]. Second, a single registry was used to explore the impact of these different definitions. External validation of the finding on a separate, ideally international, data set appears is mandatory. The authors suggest to organise an international consensus conference including resource settings and test several candidate definitions in a large international dataset. Finally, this study did not aim to recommend a single definition of THS. The objective was to provide useful methodological and conceptual insight to inform an international consensus process. Third and last, the authors acknowledge that alternative definitions could have been included in the registry-based study (a single SBP threshold, the shock index or the ATLS definition). The authors, however, believe that providing more definitions carries the risk to generate more noise without providing any gain in the main message. Namely, that THS definitions are heterogenous in the literature and how different definitions have a different impact on patient outcomes. Similarly, other THS-related parameters could have been considered in the survey such as those able to explain individual sensitivity to haemorrhage like age, comorbidities or concomitant TBI. We believe that these parameters could be of interest in further studies.

## Conclusion

This study provides a structured approach of the conundrum to find of definition of THS. It illustrates the great heterogeneity in the existing literature, from definition to outcome and highlights the difficulty to benchmarking patient groups from one study to another. The need to standardize the definition of THS persists. Any consensual candidate definition needs to be tested in large international cohorts before being adopted.

## Supplementary Information

Below is the link to the electronic supplementary material.

Supplementary file1 (DOCX 35 KB)

Supplementary file2 (DOCX 19 KB)

Supplementary file3 (DOCX 27 KB)

Supplementary file4 (DOCX 45 KB)

## Data Availability

The datasets used and/or analysed during the current study are available from the corresponding author on reasonable request.
