# Supplementary material for: The conundrum of the definition of haemorrhagic shock: a pragmatic exploration based on a scoping review, experts’ survey and a cohort analysis
Source: Eur J Trauma Emerg Surg. 2022 Jun 22;48(6):4639–49. doi: 10.1007/s00068-022-01998-9 (PMC9712310; doi:10.1007/s00068-022-01998-9)
Supplement: Supplementary file 1 — Supplementary file1 (DOCX 35 KB) [file 68_2022_1998_MOESM1_ESM.docx]

## Supplementary material 4: Thresholds proposed by the experts during the survey

| **Definition component** | **Median Score** | **Threshold** |
| --- | --- | --- |
| Base excess (mEq/L) | 2.5 | -5 |
| Blood lactate (mmol/L) | 2.0 | 2 |
| Systolic blood pressure (mmHg) | 2.0 | 90 |
| Shock index | 2.0 | 1 |
| Heart rate (beats/mn) | 1.0 | 110 |
| Response to volume expansion | 1.0 | - |
| Variation in hemoglobin (g/dL) | 1.0 | -2 |
| Haemoglobin on admission (g/dL) | 1.0 | 10 |
| Respiratory rate | 1.0 | - |
| Vasopressors | 1.0 | - |
| International Normalised Ratio | 0.5 | 1.5 |
| Mean blood pressure (mmHg) | 0.0 | 60 |
| Interventions to stop the bleeding | 0.0 | - |
| Pulse pressure (mmHg) | 0.0 | 35 |
| Capillary haemoglobin on scene (g/dL) | 0.0 | 11 |
| Viscoelastic tests | 0.0 | - |
| Capillary refill time | 0.0 | - |
| Fibrinogen concentration (g/L) | 0.0 | 1.5 |
| Mottling | 0.0 | - |
| Blood volume lost | -1.0 | - |
| Treatment response | -1.0 | - |
| Injury mechanisms | -1.0 | - |
| Platelet count (x10^3^/mm^3^) | -1.0 | 100 |
| Injury location | -1.0 | - |
| Prothrombine Time (%) | -1.5 | 50 |
| Activated Partial Thromboplastin Time | -2.0 | - |

Median score and threshold are based on experts’ responses during the online survey.
