# Supplementary material for: The conundrum of the definition of haemorrhagic shock: a pragmatic exploration based on a scoping review, experts’ survey and a cohort analysis
Source: Eur J Trauma Emerg Surg. 2022 Jun 22;48(6):4639–49. doi: 10.1007/s00068-022-01998-9 (PMC9712310; doi:10.1007/s00068-022-01998-9)
Supplement: Supplementary file 2 — Supplementary file2 (DOCX 19 KB) [file 68_2022_1998_MOESM2_ESM.docx]

## Supplementary material 1: Search equation

**Medline search equation**

1. Polytrauma*[tiab]
2. Trauma*[tiab]
3. “Multiple Trauma”[mesh]
4. hemorrhagic shock[tiab]
5. haemorrhagic shock[tiab]
6. “Shock, Hemorrhagic”[mesh]
7. animals[mh] NOT humans[mh]
8. #1 OR #2 OR #3
9. #4 OR #5 OR #6
10. #8 AND #9
11. #10 NOT #7

**Google Scholar search equation**

Trauma* AND “haemorrhagic shock”
