# Supplementary material for: The conundrum of the definition of haemorrhagic shock: a pragmatic exploration based on a scoping review, experts’ survey and a cohort analysis
Source: Eur J Trauma Emerg Surg. 2022 Jun 22;48(6):4639–49. doi: 10.1007/s00068-022-01998-9 (PMC9712310; doi:10.1007/s00068-022-01998-9)
Supplement: Supplementary file 3 — Supplementary file3 (DOCX 27 KB) [file 68_2022_1998_MOESM3_ESM.docx]

## Supplementary material 2: components used for the definition used in the cohort study

- **PROPPR study**

1. At least 1 RBC during prehospital care
2. At least 1 RBC in trauma bay (before going to CT-scan or Operating Room)
3. ABC score ≥ 2
4. a. **OR** b
   - FINAL DEFINITION : c. **AND** d.

- **PROMMTT study**

1. At least 1 RBC during prehospital care
2. At least 1 RBC in trauma bay (before going to CT-scan or Operating Room)
3. At least 4 RBC within 6 first hours in hospital
   - FINAL DEFINITION : a. **OR** b. **OR** c.

- **The Traumabase definition**

1. At least 4 RBC within 6 first hours in hospital
   - FINAL DEFINITION : a.

- **Experts based definition**

1. Prehospital Shock Index ≥ 1.0
2. Shock Index at hospital arrival ≥ 1.0
3. Base excess ≤ -.5mEq/L at hospital arrival
4. a **OR** b
   - FINAL DEFINITION : c. **AND** d.

- **Most cited definition**

1. Minimal SBP during prehospital care ≤ 70 mmHg **or** SBP 71-90 mmHg if HR ≥108/bpm
2. First SBP during prehospital care ≤ 70 mmHg **or** SBP 71-90 mmHg if HR ≥108/bpm
3. First SBP at hospital arrival ≤ 70 mmHg **or** SBP 71-90 mmHg if HR ≥108/bpm
4. Vasopressor during prehospital care
   - FINAL DEFINITION : a. **OR** b. **OR** c. **OR** d.
