# Supplementary material for: The conundrum of the definition of haemorrhagic shock: a pragmatic exploration based on a scoping review, experts’ survey and a cohort analysis
Source: Eur J Trauma Emerg Surg. 2022 Jun 22;48(6):4639–49. doi: 10.1007/s00068-022-01998-9 (PMC9712310; doi:10.1007/s00068-022-01998-9)
Supplement: Supplementary file 4 — Supplementary file4 (DOCX 45 KB) [file 68_2022_1998_MOESM4_ESM.docx]

## Supplementary material 3: definition used after standardization

| **Authors** | **Title** | **NCT if Clinical trial only** | **Year** | **Journal if published** | **Definition as extracted in the article** | **Standalone definition** |
| --- | --- | --- | --- | --- | --- | --- |
| Naumann | Safety and feasibility of sublingual microcirculation assessment in the emergency department for civilian and military patients with traumatic haemorrhagic shock: a prospective cohort study |  | 2016 | BMJ Open | (1) Injury mechanism consistent with blood loss;  (2) The patient is intubated and ventilated;  (3) Serum lactate concentration >2mmol/L;  (4) The patient has received any blood products during initial resuscitation. | (1) Injury mechanism consistent with blood loss;  (2) The patient is intubated and ventilated;  (3) Serum lactate concentration >2mmol/L;  (4) The patient has received any blood products during initial resuscitation. |
| Hutchings | Observational study of the effects of traumatic injury, haemorrhagic shock and resuscitation on the microcirculation: a protocol for the MICROSHOCK study |  | 2015 | BMJ Open | 1. Mechanism of injury consistent with blood loss; 2. Intubated and ventilated; 3. Serum lactate concentration >2 mmol/L recorded at any stage prior to admission to the ICU; 4. Have received any blood products (eg, packed red blood cells (PRBC), fresh frozen plasma (FFP), cryo- precipitate, platelets) during the initial period of resuscitation, prior to admission to ICU, or are predicted to receive blood products during this time- frame in the opinion of the trauma team leader. | 1. Mechanism of injury consistent with blood loss; 2. Intubated and ventilated; 3. Serum lactate concentration >2 mmol/L recorded at any stage prior to admission to the ICU; 4. Have received any blood products (eg, packed red blood cells (PRBC), fresh frozen plasma (FFP), cryo- precipitate, platelets) during the initial period of resuscitation, prior to admission to ICU, or are predicted to receive blood products during this time- frame in the opinion of the trauma team leader. |
|  | A Study to Evaluate the Safety and Tolerability of Valproic Acid in Trauma Patients(Part 2) |  |  |  | Two or more systolic blood pressure readings of ≤ 110 mmHg at any point during transport to the hospital or within the first hour after arrival in the emergency department. Systolic blood pressure readings of ≤ 110 mmHg need not be consecutive | 2 or SBP <110 during prehospital care or within the first hour in TRR |
| Gauss | Effect of early use of noradrenaline on in-hospital mortality in haemorrhagic shock after major trauma: a propensity-score analysis |  | 2018 | British Journal of Anaesthesia | Transfusion of more than four erythrocyte concentrates in the first 6 h of admission | 4 CG in 6h |
| Boutonnet | Tranexamic acid in severe trauma patients managed in a mature trauma care system |  | 2018 | J Trauma Acute Care Surg | Transfusion of ≥4 erythrocyte-concentrate units during the first 6 h | 4 CG in 6h |
| Caputo | A retrospective analysis of the respiratory adjusted shock index to determine the presence of occult shock in trauma patients |  | 2018 | J Trauma Acute Care Surg | A lactate greater than 2 mmol/L was used to determine presence of hypoperfusion | A lactate greater than 2 mmol/L was used to determine presence of hypoperfusion |
| Galvagno | Outcomes after concomitant traumatic brain injury and hemorrhagic shock: A secondary analysis from the Pragmatic, Randomized Optimal Platelets and Plasma Ratios trial |  | 2017 | J Trauma Acute Care Surg | Admission base excess of −4 or less and/or admission shock index of 0.9 or greater. | Admission base excess of −4 or less and/or admission shock index of 0.9 or greater. |
| Savage | Redefining massive transfusion when every second counts |  | 2013 | J Trauma Acute Care Surg | All civilian trauma patients who received at least one unit of blood in the first 24 hours of admission | All civilian trauma patients who received at least one unit of blood in the first 24 hours of admission |
| Holcomb | Transfusion of Plasma, Platelets, and Red Blood Cells in a 1:1:1 vs a 1:1:2 Ratio and Mortality in Patients With Severe Trauma: The PROPPR Randomized Clinical Trial |  | 2015 | JAMA | All of the Following: Highest trauma level activation ; Estimated age of 15 years or older or weight of 50 kg or greater if age unknown ; Received directly from the injury scene ; Initiated transfusion of at least 1 U of blood component within the first hour of arrival or during prehospital transport ; Predicted to receive a massive transfusion by exceeding the threshold score of either the Assessment of Blood Consumption score of 2 or greater or based on the attending trauma physician’s judgment | All of the Following: Highest trauma level activation ; Estimated age of 15 years or older or weight of 50 kg or greater if age unknown ; Received directly from the injury scene ; Initiated transfusion of at least 1 U of blood component within the first hour of arrival or during prehospital transport ; Predicted to receive a massive transfusion by exceeding the threshold score of either the Assessment of Blood Consumption score of 2 or greater or based on the attending trauma physician’s judgment |
| Guly | Testing the validity of the ATLS classification of hypovolaemic shock |  | 2010 | Resuscitation | ATLS definition of shock based on : blood loss, HR, SBP, PP, RR, Mental status. | Any ATLS class |
| Cannon JW | Hemorrhagic shock |  | 2018 | N Engl J Med | ATLS classification (and : anxiety, tachypnea, a weak peripheral pulse, and cool extremities with pale or mottled skin, base deficit, lactates values, ...) | Any ATLS class |
| Hamada | Development and validation of a pre-hospital “Red Flag” alert for activation of intra-hospital haemorrhage control response in blunt trauma |  | 2018 | Critical Care | Any of the following criteria was present: need for any packed (RBC) transfusion upon arrival in the resuscitation room, transfusion of 4 packed RBCs or more within the first 6 h, blood lactate concentration ≥ 5 mmol/L upon arrival, need for immediate haemostatic surgery or interventional radiology before complete injury assessment by whole-body CT scan or death from haemorrhagic shock | Any of the following criteria was present: need for any packed red blood cell (RBC) transfusion upon arrival in the resuscitation room, transfusion of 4 packed RBCs or more within the first 6 h, blood lactate concentration ≥ 5 mmol/L upon arrival, need for immediate haemostatic surgery or interventional radiology before complete injury assessment by whole-body CT scan or death from haemorrhagic shock |
| Stanworth | Mortality from trauma haemorrhage and opportunities for improvement in transfusion practice. |  | 2016 | Br J Surg | At least 4 units of packed red blood cells (PRBCs) in the first 24 h of admission with activation of the massive haemorrhage protocol | At least 4 units of packed red blood cells (PRBCs) in the first 24 h of admission with activation of the massive haemorrhage protocol |
| Morrison | Intra‐operative correction of acidosis, coagulopathy and hypothermia in combat casualties with severe haemorrhagic shock |  | 2013 | Anesthesia | At least one major (proximal to ankle) leg amputation, and required laparotomy (for abdominal injury or proximal control of torn leg arteries) | At least one major (proximal to ankle) leg amputation, and required laparotomy (for abdominal injury or proximal control of torn leg arteries) |
| Rizoli | The immunomodulatory effects of hypertonic saline resuscitation in patients sustaining traumatic hemorrhagic shock: a randomized, controlled, double-blinded trial. Saline Resuscitation in Patients Sustaining Traumatic Hemorrhagic Shock |  | 2006 | Ann Surg | At least one recorded episode of hypotension (systolic blood pressure 90 mm Hg) with clear evidence of blood loss (external or internal including thorax, abdomen, or retroperitoneum) | At least one recorded episode of hypotension (systolic blood pressure 90 mm Hg) with clear evidence of blood loss (external or internal including thorax, abdomen, or retroperitoneum) |
|  | REVIVE: Reducing Exsanguination Via In‐Vivo Expandable Foam | NCT02880163 |  |  | ATLS class 3 or 4 or ABC ≥ 2 (abdominal), Subject must also be receiving concurrent transfusion of fluids or blood products. | ATLS class 3 or 4 or ABC ≥ 2 (abdominal), Subject must also be receiving concurrent transfusion of fluids or blood products. |
| Lawton | The utility of Advanced Trauma Life Support (ATLS) clinical shock grading in assessment of trauma |  | 2014 | Emergency Medicine journal | ATLS classification > class 0 | ATLS classification > class 0 |
|  | Efficacy, Safety of Solution Containing Hyperosmolar Sodium Lactate Infusion for Resuscitation of Patients With Hemorrhagic Shock | NCT01433276 |  |  | Grade III hemorrhagic shock (who fulfilled at least 2 of these following criteria: 1.5-2 L blood loss estimation, MAP <65 mmHg, pulse pressure <20 mmHg, heart rate >120 times/min, respiratory rate 30-40 times/min or urinary output 5-15 ml/hour) due to multiple injuries (simultaneously injuries in two or more organs of the body), Survival probability is >50%, predicted by Revised Trauma Score ≥ 4 (scale 0-7.8408) | ATLS grade III hemorrhagic shock (who fulfilled at least 2 of these following criteria: 1.5-2 L blood loss estimation, MAP <65 mmHg, pulse pressure <20 mmHg, heart rate >120 times/min, respiratory rate 30-40 times/min or urinary output 5-15 ml/hour) due to multiple injuries (simultaneously injuries in two or more organs of the body), Survival probability is >50%, predicted by Revised Trauma Score ≥ 4 (scale 0-7.8408) |
|  | Shock, Whole Blood, and Assessment of TBI S.W.A.T. (LITES TO 2) | NCT03402035 |  |  | Patients with blunt or penetrating injury who meet the following criteria: 1, 2, and 3 Has 2 or more of any of the following: Hypotension (systolic blood pressure ≤ 90 mmHg) in the prehospital or emergency department setting, Penetrating mechanism, Positive FAST abdominal ultrasound, Heart Rate ≥ 120 in the prehospital or emergency department setting. AND Taken to the Operating Room (laparotomy, thoracotomy or vascular exploration) or Interventional Radiology within 60 minutes of arrival. AND Need of blood/blood component transfusion in prehospital setting, ED or OR within 60 minutes of arrival. | At least 2 agmong : SBP <90 during prehospital cares (or in ED) or penetrating or positive e abdominal FAST +or HR > 120during pre hospital care (or in ED)  AND surgery or RI  AND Transfusion |
| Cole | Tranexamic Acid Use in Severely Injured Civilian Patients and the Effects on Outcomes |  | 2015 | Ann Surg | Base Deficit ≥ 6 mEq/L | Base Deficit ≥ 6 mEq/L |
| Furmaga | Novel markers predict death and organ failure following hemorrhagic shock |  | 2015 | Clin Chimi Acta | blunt trauma: systolic blood pressure 90 mmHg and a shock index ranging from 0.81 to 2.33 | blunt trauma: systolic blood pressure 90 mmHg and a shock index ranging from 0.81 to 2.33 |
|  | Use of Blood and Plasma in Norwegian Physician-staffed Helicopter Emergency Medical Systems | NCT02784951 |  |  | FC>100 or absent/weak radial pulse// PAS<90/ confusion without head trauma | FC>100 or absent/weak radial pulse// PAS<90/ confusion without head trauma |
| Corradi | Hemorrhagic Shock in Polytrauma Patients: Early Detection with Renal Doppler Resistive Index Measurements |  | 2011 | radiology | Hemorrhagic shock was defined as low blood pressure (systolic blood pressure, 90 mm Hg), low urine output (30 mL/h), and a blood lactate level greater than 2 mmol/L | Hemorrhagic shock was defined as low blood pressure (systolic blood pressure ,90 mm Hg), low urine output (30 mL/h), and a blood lactate level greater than 2 mmol/L (23) |
| Pape | The definition of polytrauma revisited: An international consensus process and proposal of the new ‘Berlin definition’ |  | 2014 | The Journal of Trauma: Injury, Infection, and Critical Care | Hypotension was defined as a systolic blood pressure of 90 mm Hg or lower. Metabolic acidosis was defined as a base excess of 6 or lower.  Coagulopathy was defined as a partial thromboplastin time (PTT) of 50 or greater or an INR value of 1.4 or greater. | Hypotension was defined as a systolic blood pressure of 90 mm Hg or lower. Metabolic acidosis was defined as a base excess of 6 or lower.  Coagulopathy was defined as a partial thromboplastin time (PTT) of 50 or greater or an INR value of 1.4 or greater. |
|  | The Cutoff Point for Caval Index and Its Correlation With Central Venous Pressure and Plasma Lactate Level for Assessing Patients in Hypovolemic Hemorrhagic States | NCT01741818 |  |  | Hypovolemic hemorrhagic subjects (class II or more) | Hypovolemic hemorrhagic subjects (class II or more) |
|  | Comparison of Rapid Thrombelastography and Conventional Coagulation Testing for Haemostatic Resuscitation in Trauma | NCT01536496 |  |  | ISS>15 and RBC within h6 | ISS>15 and RBC within h6 |
|  | Retroperitoneal Packing or Angioembolization for Hemorrhage Control of Pelvic Fractures | NCT02535624 |  |  | ISS>17, Tile B or C pelvic fracture and x-Ray, SBP<90 after 4RBC | ISS>17, Tile B or C pelvic fracture and x-Ray, SBP<90 after 4RBC |
|  | Effect of Remote Ischemic Conditioning on Trauma Patients With Hemorrhagic Shock | NCT02071290 |  |  | at least one SBP≤90 / identified source of blood loss or blood products ordered to the trauma room/<3h | at least one SBP≤90 / identified source of blood loss or blood products ordered to the trauma room/<3h |
|  | Resuscitative Endocrinology: Single-dose Clinical Uses for Estrogen - Traumatic Hemorrhagic Shock (RESCUE - Shock) | NCT00973102 |  |  | Pre-hospital or ED systolic blood pressure < 90 | SBP 90 |
|  | Vasopressin Deficiency in Hemorrhagic Shock | NCT01107314 |  |  | reported or actual systolic blood pressure < 90 mmHg within 1 hour of arrival to the Emergency Room | SBP 90 |
|  | Field Trial of Hypotensive Versus Standard Resuscitation for Hemorrhagic Shock After Trauma | NCT01411852 |  |  | pre-hospital SBP ≤ 90 mmHg | SBP 90 |
|  | The Trauma- Formula-Driven Versus Lab-Guided Study (TRFL Study) | NCT00945542 |  |  | SBP HS mais expected to need MT: bleeding and expected to require massive transfusion (either 4 units within the next 2 hours or ≥ 10 units of RBC in 24 h) or required transfusion of un-cross matched emergency stock red blood cells; and had an episode of hypotension (systolic bp ≤ 90mmHg). | SBP HS but expected to need MT: bleeding and expected to require massive transfusion (either 4 units within the next 2 hours or ≥ 10 units of RBC in 24 h) or required transfusion of un-cross matched emergency stock red blood cells; and had an episode of hypotension (systolic bp ≤ 90mmHg). |
|  | California Prehospital and In Hospital Antifibrinolytic Therapy Via TXA | NCT03469947 |  |  | SBP <90 pre-hosp ou dechoc ou a risque significant hemorrage: Estimated blood loss of 500 milliliters int he field accompanied with HR >120/ Bleeding not controlled by direct pressure or tourniquet/Major amputation of any extremity above the wrists and above the ankles | SBP <90 pre-hosp ou dechoc ou a risque significant hemorrage: Estimated blood loss of 500 milliliters int he field accompanied with HR >120/ Bleeding not controlled by direct pressure or tourniquet/Major amputation of any extremity above the wrists and above the ankles |
|  | Use of Nitroglycerine to Improve Signs of Poor Peripheral Perfusion in Patients With Traumatic Hemorrhagic Shock | NCT03235921 |  |  | SBP <90, mean blood pressure below70mmhg or decrease of systolic blood pressure 40mmhg below normal value. PH<7,35 due ti hypoperfusion, Capillary refill time > 4 seconds. | PAS<90, mean blood pressure below 70mmhg or decrease of systolic blood pressure 40mmhg below normal value. PH<7,35 due to hypoperfusion, Capillary refill time > 4 seconds. |
| Jenkins | Trauma hemostasis and oxygenation research position paper on remote damage control resuscitation: definitions, current practice, and knowledge gaps |  | 2014 | Shock | pathophysiological state that occurs when oxygen delivery is insufficient to maintain aerobic respiration in tissue | pathophysiological state that occurs when oxygen delivery is insufficient to maintain aerobic respiration in tissue |
| Clarke | Time to laparotomy for intra-abdominal bleeding from trauma does affect survival for delays up to 90 minutes |  | 2002 | J Trauma | patient’s SBP was greater than 0 mm Hg and less than or equal to 90 mm Hg on arrival in the ED | patient’s SBP was greater than 0 mm Hg and less than or equal to 90 mm Hg on arrival in the ED |
|  | Philadelphia Immediate Transport in Penetrating Trauma Trial | NCT02821364 |  |  | penetrating + shock =Heart rate greater than 100, systolic blood pressure less than 100 or mental status change (evidence of shock) | penetrating + shock =Heart rate greater than 100, systolic blood pressure less than 100 or mental status change (evidence of shock) |
| Pre-Hospital Use of Plasma for Traumatic Hemorrhage |  | NCT02303964 |  |  | pre-hospital SBP <70 or 70-90 with HR >108, Ongoing hemorrhage with unstable vital signs | pre-hospital SBP <70 or 70-90 with HR >108, Ongoing hemorrhage with unstable vital signs |
| Meyer | A comparison of resuscitation intensity and critical administration threshold in predicting early mortality among bleeding patients: A multicenter validation in 680 major transfusion patients |  | 2018 | J Trauma Acute Care Surg | Resuscitation intensity was defined as total products in the first 30 minutes of arrival (1 U RBC, 1 U plasma, 1000 mL crystalloid, 500 mL colloid each assigned a value of 1) | Resuscitation intensity was defined as total products in the first 30 minutes of arrival (1 U RBC, 1 U plasma, 1000 mL crystalloid, 500 mL colloid each assigned a value of 1) |
| Gonzales | Goal directed hemostatic resuscitation of trauma induced coagulopathy |  | 2016 | Ann Surg | Resuscitation Outcome Consortium criteria [systolic blood pressure (SBP) <70 mm Hg or SBP 70–90 mm Hg with heart rate (HR) ≥108 beats/min], in addition to any of the following injury patterns: penetrating torso wound, unstable pelvic fracture, or abdominal ultrasound suspicious of bleeding in more than one region | Resuscitation Outcome Consortium criteria [systolic blood pressure (SBP) <70 mm Hg or SBP 70–90 mm Hg with heart rate (HR) ≥108 beats/min], in addition to any of the following injury patterns: penetrating torso wound, unstable pelvic fracture, or abdominal ultrasound suspicious of bleeding in more than one region |
|  | HMGB1 Release From Hemorrhagic Shock Patients | NCT03535441 |  |  | out-of-hospital systolic blood pressure (SBP) of 70 mmHg or less or SBP ranging 71 to 90 mmHg with a heart rate of 108 beats/min or more. | SBP < 90mmHg with tachycardia >108 OR SBP<70 without the tachycardia requirement |
|  | Pragmatic Prehospital Group O Whole Blood Early Resuscitation Trial | NCT03477006 |  |  | medical aur service: hypotension (SBP < 90mmHg with tachycardia >108 OR SBP<70 without the tachycardia requirement) | SBP < 90mmHg with tachycardia >108 OR SBP<70 without the tachycardia requirement |
|  | Control of Major Bleeding After Trauma Study | NCT01838863 |  |  | pre-hosp: SBP<70 mmHg or SBP 71-90 mmHg with heart rate (HR)>108 beats per minute. | SBP < 90mmHg with tachycardia >108 OR SBP<70 without the tachycardia requirement |
| Chapman | COMBAT: Initial experience with a randomized clinical trial of plasma-based resuscitation in the field for traumatic hemorrhagic shock |  | 2015 | Shock | traumatically injured adults with either a systolic blood pressure (SBP) ≤ 70 mmHg or 71-90 mmHg with an accompanying heart rate ≥108. | SBP < 90mmHg with tachycardia >108 OR SBP<70 without the tachycardia requirement |
| Delano | Prehospital Resuscitation of Traumatic Hemorrhagic Shock with Hypertonic Solutions Worsens Hypocoagulation and Hyperfibrinolysis |  | 2015 | Shock | out-of-hospital systolic blood pressure (SBP) of 70 mmHg or less or SBP 71 to 90 mmHg with a heart rate of 108 beats/min or more | SBP < 90mmHg with tachycardia >108 OR SBP<70 without the tachycardia requirement |
| Bulger | Out-of-hospital hypertonic resuscitation after traumatic hypovolemic shock: a randomized, placebo controlled trial |  | 2011 | Ann Surg | out-of-hospital systolic blood pressure (SBP) 70 mm Hg or less or 71 to 90 mm Hg with a concomitant heart rate (HR) 108 beats or less per minute | SBP < 90mmHg with tachycardia >108 OR SBP<70 without the tachycardia requirement |
| Moore | Plasma First in the Field for Postinjury Hemorrhagic Shock |  | 2014 | Shock | acute blood loss with systolic blood pressure (SBP) less than 70 mmHg or SPB 71 to 90 mmHg with a heart rate greater than 108 beats/min | SBP < 90mmHg with tachycardia >108 OR SBP<70 without the tachycardia requirement |
| Wang | Association of out-of-hospital advanced airway management with outcomes after traumatic brain injury and hemorrhagic shock in the ROC hypertonic saline trial |  | 2014 | Emergency Medicine journal | systolic blood pressure of ≤70 mm Hg, or a systolic blood pressure of 71–90 mm Hg with a concomitant heart rate ≥108 beats per minute | SBP < 90mmHg with tachycardia >108 OR SBP<70 without the tachycardia requirement |
| Moore | Plasma-first resuscitation to treat haemorrhagic shock at the scene of injury and during emergency ground transportation in an urban area: a randomised trial |  | 2018 | Lancet | systolic blood pressure [SBP] ≤70 mm Hg or 71–90 mm Hg plus heart rate ≥108 beats per min | SBP < 90mmHg with tachycardia >108 OR SBP<70 without the tachycardia requirement |
| Sperry | Prehospital Plasma during Air Medical Transport in Trauma Patients at Risk for Hemorrhagic Shock |  | 2018 | N Engl J Med | at least one episode of hypotension (systolic blood pressure <90 mm Hg) and tachycardia (defined in this trial as a heart rate >108 beats per minute) or if they had any severe hypotension (systolic blood pressure <70 mm Hg), either before the arrival of air medical transport or any time before arrival at the trauma center | SBP < 90mmHg with tachycardia >108 OR SBP<70 without the tachycardia requirement |
| Brasel | Hypertonic Resuscitation: Design and Implementation of a Prehospital Intervention Trial |  | 2008 | JACS | SBP 70 mmHg alone or those with an SBP 71 to 90 mmHg and a heart rate 108 beats per minute | SBP < 90mmHg with tachycardia >108 OR SBP<70 without the tachycardia requirement |
| Junger | Resuscitation of traumatic hemorrhagic shock patients with hypertonic saline - without dextran - inhibits neutrophil and endothelial cell activation |  | 2012 | Shock | prehospital systolic blood pressure (SBP) of 70 mmHg or less and patients who had an SBP between 71 and 90 mmHg as well as a concomitant heart rate of 108 beats/min or greater. | SBP < 90mmHg with tachycardia >108 OR SBP<70 without the tachycardia requirement |
| Olaussen | Review article: Shock Index for prediction of critical bleeding post‐trauma: A systematic review |  | 2014 | Emergency Medicine Australalia | Shock index | Shock index |
| Woolley | Trauma Hemostasis and Oxygenation Research Network position paper on the role of hypotensive resuscitation as part of remote damage control resuscitation |  | 2018 | J Trauma Acute Care Surg | Shock is a state of oxygen delivery that is inadequate to meet vital organ metabolic demands. | Shock is a state of oxygen delivery that is inadequate to meet vital organ metabolic demands. |
|  | Safety and Efficacy of PolyHeme(R) in Hemorrhagic Shock Following Traumatic Injuries Beginning in the Pre-Hospital Setting | NCT00076648 |  |  | sustained blood loss and are in shock: field PAS<90 | sustained blood loss and are in shock: field PAS<90 |
| Tachon | Microcirculatory Alterations in Traumatic Hemorrhagic Shock |  | 2014 | Critical Care Medicine | systolic arterial pressure less than or equal to 90mm Hg associated with greater than or equal to 4 packed RBCs within 6 hours of hospital care | systolic arterial pressure less than or equal to 90mm Hg associated with greater than or equal to 4 packed RBCs within 6 hours of hospital care |
|  | Pre-hospital Administration of Lyophilized Plasma for Post-traumatic Coagulopathy Treatment (PREHO-PLYO) | NCT02736812 |  |  | Systolic Blood Pressure <70 mmHg OR [between 71 and 90 mmHg AND heart rate > 108] at some point in the medicalized care] OR [Shock index > 1.3] | Systolic Blood Pressure <70 mmHg OR [between 71 and 90 mmHg AND heart rate > 108] at some point in the medicalized care] OR [Shock index > 1.3] |
| Irahara | Retrospective study of the effectiveness of Intra-Aortic Balloon Occlusion (IABO) for traumatic haemorrhagic shock |  | 2015 | World Journal of Emergency Surgery | systolic blood pressure <90 mmHg or shock index ≥1 | systolic blood pressure <90 mmHg or shock index ≥1 |
| Khan | The role of pharmacological steroid therapy in preservation of renal function in severely injured patients requiring massive transfusion |  | 2016 | Eur J Trauma Emerg Surg | systolic blood pressure less than 80 mm of Hg with an obvious need for operative intervention | Systolic blood pressure less than 80 mm of Hg with an obvious need for operative intervention |
| Maegele | Revalidation and update of the TASH-Score: a scoring system to predict the probability for massive transfusion as a surrogate for life-threatening haemorrhage after severe injury |  | 2011 | Vox Sanguinis | TASH score | TASH score |
| Callaway | Serum lactate and base deficit as predictors of mortality in normotensive elderly blunt trauma patients |  | 2009 | J Trauma | the following criteria: (1) initial systolic blood pressure ≤ 90 mm Hg (upon arrival to the ED); (2) BD or a lactate measured at admission to the ED; and (3) blunt mechanisms of trauma | the following criteria: (1) initial systolic blood pressure ≤ 90 mm Hg (upon arrival to the ED); (2) BD or a lactate measured at admission to the ED; and (3) blunt mechanisms of trauma |
| Butler | Fluid Resuscitation in Tactical Combat Casualty Care: Yesterday and Today |  | 2017 | Wilderness Environ Med | The tactically relevant definition of shock was therefore identified as: 1) unconsciousness or altered mental status (confused or drowsy) that is not due to TBI or drug therapy; and/or 2) abnormal (ie, weak or absent) radial pulse. | The tactically relevant definition of shock was therefore identified as: 1) unconsciousness or altered mental status (confused or drowsy) that is not due to TBI or drug therapy; and/or 2) abnormal (ie, weak or absent) radial pulse.35 |
|  | PROCOAG: Early Administration of Prothrombin Concentrate Complex in Patients With Acute Hemorrhage Following Severe Trauma | NCT03218722 |  |  | Pre hospital RBC transfusion or during the first hour after hospital admission or ABC score≥2 or massive transfusion expected by the attending physician | Pre hospital RBC transfusion or during the first hour after hospital admission or ABC score≥2 or massive transfusion expected by the attending physician |
| Holcomb | The Prospective, Observational, Multicenter, Major Trauma Transfusion (PROMMTT) Study: Comparative Effectiveness of a Time-varying Treatment with Competing Risks |  | 2013 | JAMA Surg | transfusion of at least 1 unit of RBCs in the first 6 hours after admission | transfusion of at least 1 unit of RBCs in the first 6 hours after admission |
| Mitra | The definition of massive transfusion in trauma: a critical variable in examining evidence for resuscitation |  | 2011 | Eur J Emerg Med | transfusion of at least 5 units in 4 h | transusion of at least 5 units in 4 h |
|  | Vasopressin in Traumatic Hemorrhagic Shock Study | NCT00379522 |  |  | traumatic hemorrhagic shock (systolic arterial blood pressure <90 mm Hg) that does not respond to the first 10 min of standard shock treatment [endotracheal intubation, crystalloid-, colloid-, and hypertonic saline (up to 4 ml/kg) fluid resuscitation, and catecholamine (ephedrine, phenylephrine, norepinephrine, epinephrine) vasopressors]. | traumatic hemorrhagic shock (systolic arterial blood pressure <90 mm Hg) that does not respond to the first 10 min of standard shock treatment [endotracheal intubation, crystalloid-, colloid-, and hypertonic saline (up to 4 ml/kg) fluid resuscitation, and catecholamine (ephedrine, phenylephrine, norepinephrine, epinephrine) vasopressors]. |
| Morrison | Hypotensive Resuscitation Strategy Reduces Transfusion Requirements and Severe Postoperative Coagulopathy in Trauma Patients With Hemorrhagic Shock: Preliminary Results of a Randomized Controlled Trial |  | 2011 | J Trauma | undergoing laparotomy or thoracotomy for blunt and penetrating trauma who had at least one in-hospital documented systolic blood pressure (SBP) 90 mm Hg | Undergoing laparotomy or thoracotomy for blunt and penetrating trauma who had at least one in-hospital documented systolic blood pressure (SBP) 90 mm Hg |
| Smith | Randomized Controlled Trial Evaluating the Efficacy of Peritoneal Resuscitation in the Management of Trauma Patients Undergoing Damage Control Surgery Management of Trauma Patients Undergoing Damage Control Surgery |  | 2017 | J Am Coll Surg | within 4 hours of hospital admission by the presence of 3 of the following: 1)Tachycardia (>120 beats/min); 2) hypotension (Systolic BP <90 mmHg or initiation of initiation of vasopressor Rx); 3) global hypoperfusion (pH<7.32, BD<-4, Serum Lactate>3.0, SvO2<60%); 4) oliguria (Urine Output < 0.5 cc/kg/hr for 2 hours); and/or 5) blood transfusion requirement of >4 units in the initial 2 hrs. post admission | Within 4 hours of hospital admission by the presence of 3 of the following: 1)Tachycardia (>120 beats/min); 2) hypotension (Systolic BP <90 mmHg or initiation of initiation of vasopressor Rx); 3) global hypoperfusion (pH<7.32, BD<-4, Serum Lactate>3.0, SvO2<60%); 4) oliguria (Urine Output < 0.5 cc/kg/hr for 2 hours); and/or 5) blood transfusion requirement of >4 units in the initial 2 hrs. post admission |
